# Supplementary material for: A comprehensive survey of C. elegans argonaute proteins reveals organism-wide gene regulatory networks and functions
Source: eLife. 2023 Feb 15;12:e83853. doi: 10.7554/eLife.83853 (PMC10101689; doi:10.7554/eLife.83853)
Supplement: Figure 1—figure supplement 1—source data 1. [file elife-83853-fig1-figsupp1-data1.zip › Figure S1/Figure S1 Blots Legend.docx]

A.

Original File: A_GFP-3xFLAG_ALG-1_ALG-2.tif

GFP::3xFLAG::ALG-1 IP (Chromotek anti-GFP nanobodies)/WB (mouse anti-FLAG M2 antibody)

GFP::3xFLAG::ALG-2 IP (Chromotek anti-GFP nanobodies)/WB (mouse anti-FLAG M2 antibody)

Blot from Figure S1B

Synchronized YA hermaphrodite samples

In = Input (total lysate) 100ug

IP = Immunoprecipitation with anti-GFP beads 10% of 5mg IP

M = Immunoprecipitation with non-specific antibody beads 10% of 5mg IP

MW of GFP:3xFLAG::ALG-1 ~ 140kDa

MW of GFP:3xFLAG::ALG-2 ~ 140kDa

B.

Original File: B_GFP-3xFLAG_ALG-3_ALG-5_ERGO-1.tif

GFP::3xFLAG::ALG-3 IP (Chromotek anti-GFP nanobodies)/WB (mouse anti-FLAG M2 antibody)

GFP::3xFLAG::ALG-5 IP (Chromotek anti-GFP nanobodies)/WB (mouse anti-FLAG M2 antibody)

GFP::3xFLAG::ERGO-1 IP (Chromotek anti-GFP nanobodies)/WB (mouse anti-FLAG M2 antibody)

Blot from Figure S1B

Synchronized L4 hermaphrodite samples (ALG-3)

Synchronized YA hermaphrodite samples (ALG-5, ERGO-1)

In = Input (total lysate) 100ug

IP = Immunoprecipitation with anti-GFP beads 10% of 5mg IP

M = Immunoprecipitation with non-specific antibody beads 10% of 5mg IP

MW of GFP:3xFLAG::ALG-3 ~ 140kDa

MW of GFP:3xFLAG::ALG-5 ~ 140kDa

MW of GFP:3xFLAG::ERGO-1 ~ 160kDa

C.

Original File: C_GFP-3xFLAG_ALG-3_ALG-4_L4.tif

Original File: C_GFP-3xFLAG_ALG-3_ALG-4_L4_2.tif

GFP::3xFLAG::ALG-4 IP (Chromotek anti-GFP nanobodies)/WB (mouse anti-FLAG M2 antibody)

Blot from Figure S1B

Synchronized L4 hermaphrodite samples

In = Input (total lysate) 100ug

IP = Immunoprecipitation with anti-GFP beads 10% of 5mg IP

M = Immunoprecipitation with non-specific antibody beads 10% of 5mg IP

MW of GFP:3xFLAG::ALG-4 ~ 140kDa

D.

Original File: D_GFP-3xFLAG_RDE-1_CSR-1_WAGO-1.tif

GFP::3xFLAG::RDE-1 IP (Chromotek anti-GFP nanobodies)/WB (mouse anti-FLAG M2 antibody)

GFP::3xFLAG::CSR-1 IP (Chromotek anti-GFP nanobodies)/WB (mouse anti-FLAG M2 antibody)

GFP::3xFLAG::WAGO-1 IP (Chromotek anti-GFP nanobodies)/WB (mouse anti-FLAG M2 antibody)

Blot from Figure S1B

Synchronized YA hermaphrodite samples

In = Input (total lysate) 100ug (20ug for CSR-1)

IP = Immunoprecipitation with anti-GFP beads 10% of 5mg IP

M = Immunoprecipitation with non-specific antibody beads 10% of 5mg IP

MW of GFP::3xFLAG::RDE-1 ~ 150kDa

MW of GFP::3xFLAG::CSR-1 ~ 130kDa

MW of GFP::3xFLAG::WAGO-1 ~ 140kDa

E.

Original File: E_GFP-3xFLAG_ALG-1_ALG-2_PRG-1.tif

GFP::3xFLAG::PRG-1 IP (Chromotek anti-GFP nanobodies)/WB (mouse anti-FLAG M2 antibody)

Blot from Figure S1B

Synchronized YA hermaphrodite samples

In = Input (total lysate) 100ug

IP = Immunoprecipitation with anti-GFP beads 10% of 5mg IP

M = Immunoprecipitation with non-specific antibody beads 10% of 5mg IP

MW of GFP:3xFLAG::PRG-1 ~ 130kDa

F.

Original File: F_GFP-3xFLAG_C04F12.1_RDE-1.tif

GFP::3xFLAG::C04F12.1/VSRA-1 IP (Chromotek anti-GFP nanobodies)/WB (mouse anti-FLAG M2 antibody)

Blot from Figure S1B

Synchronized YA hermaphrodite samples

In = Input (total lysate) 100ug

IP = Immunoprecipitation with anti-GFP beads 10% of 5mg IP

M = Immunoprecipitation with non-specific antibody beads 10% of 5mg IP

MW of GFP:3xFLAG::C04F12.1/VSRA-1 ~ 130kDa

G.

Original File: G_GFP-3xFLAG-PPW-2_WAGO-4_HRDE-1.tif

GFP::3xFLAG::PPW-2 IP (Chromotek anti-GFP nanobodies)/WB (mouse anti-FLAG M2 antibody)

GFP::3xFLAG::WAGO-4 IP (Chromotek anti-GFP nanobodies)/WB (mouse anti-FLAG M2 antibody)

GFP::3xFLAG::HRDE-1 IP (Chromotek anti-GFP nanobodies)/WB (mouse anti-FLAG M2 antibody)

Blot from Figure S1B

Synchronized YA hermaphrodite samples

In = Input (total lysate) 100ug

IP = Immunoprecipitation with anti-GFP beads 10% of 5mg IP

M = Immunoprecipitation with non-specific antibody beads 10% of 5mg IP

MW of GFP::3xFLAG::PPW-2 ~ 130kDa

MW of GFP::3xFLAG::WAGO-4 ~ 140kDa

MW of GFP::3xFLAG::HRDE-1 ~ 130kDa

H.

Original File: H_GFP-3xFLAG_PPW-1_SAGO-2_SAGO-1_2.tif

GFP::3xFLAG::PPW-1 IP (Chromotek anti-GFP nanobodies)/WB (mouse anti-FLAG M2 antibody)

GFP::3xFLAG::SAGO-2 IP (Chromotek anti-GFP nanobodies)/WB (mouse anti-FLAG M2 antibody)

GFP::3xFLAG::SAGO-1 IP (Chromotek anti-GFP nanobodies)/WB (mouse anti-FLAG M2 antibody)

Blot from Figure S1B

Synchronized YA hermaphrodite samples

In = Input (total lysate) 100ug

IP = Immunoprecipitation with anti-GFP beads 10% of 5mg IP

M = Immunoprecipitation with non-specific antibody beads 10% of 5mg IP

MW of GFP::3xFLAG::PP1-2 ~ 130kDa

MW of GFP::3xFLAG::SAGO-2 ~ 130kDa

MW of GFP::3xFLAG::SAGO-1 ~ 130kDa

I-J.

Original File: I_J_GFP-3xFLAG_WAGO-10_NRDE-3.tif

GFP::3xFLAG::WAGO-10 IP (Chromotek anti-GFP nanobodies)/WB (mouse anti-FLAG M2 antibody)

GFP::3xFLAG::NRDE-3 IP (Chromotek anti-GFP nanobodies)/WB (mouse anti-FLAG M2 antibody)

Blot from Figure S1B

Synchronized L4 hermaphrodite samples (WAGO-10)

Synchronized YA hermaphrodite samples (NRDE-3)

In = Input (total lysate) 100ug

IP = Immunoprecipitation with anti-GFP beads 10% of 5mg IP

M = Immunoprecipitation with non-specific antibody beads 10% of 5mg IP

MW of GFP:3xFLAG::WAGO-10 ~ 150kDa

MW of GFP:3xFLAG::NRDE-3 ~ 150kDa
