# Supplementary figures and images for: A comprehensive survey of C. elegans argonaute proteins reveals organism-wide gene regulatory networks and functions
Source: eLife. 2023 Feb 15;12:e83853. doi: 10.7554/eLife.83853 (PMC10101689; doi:10.7554/eLife.83853)

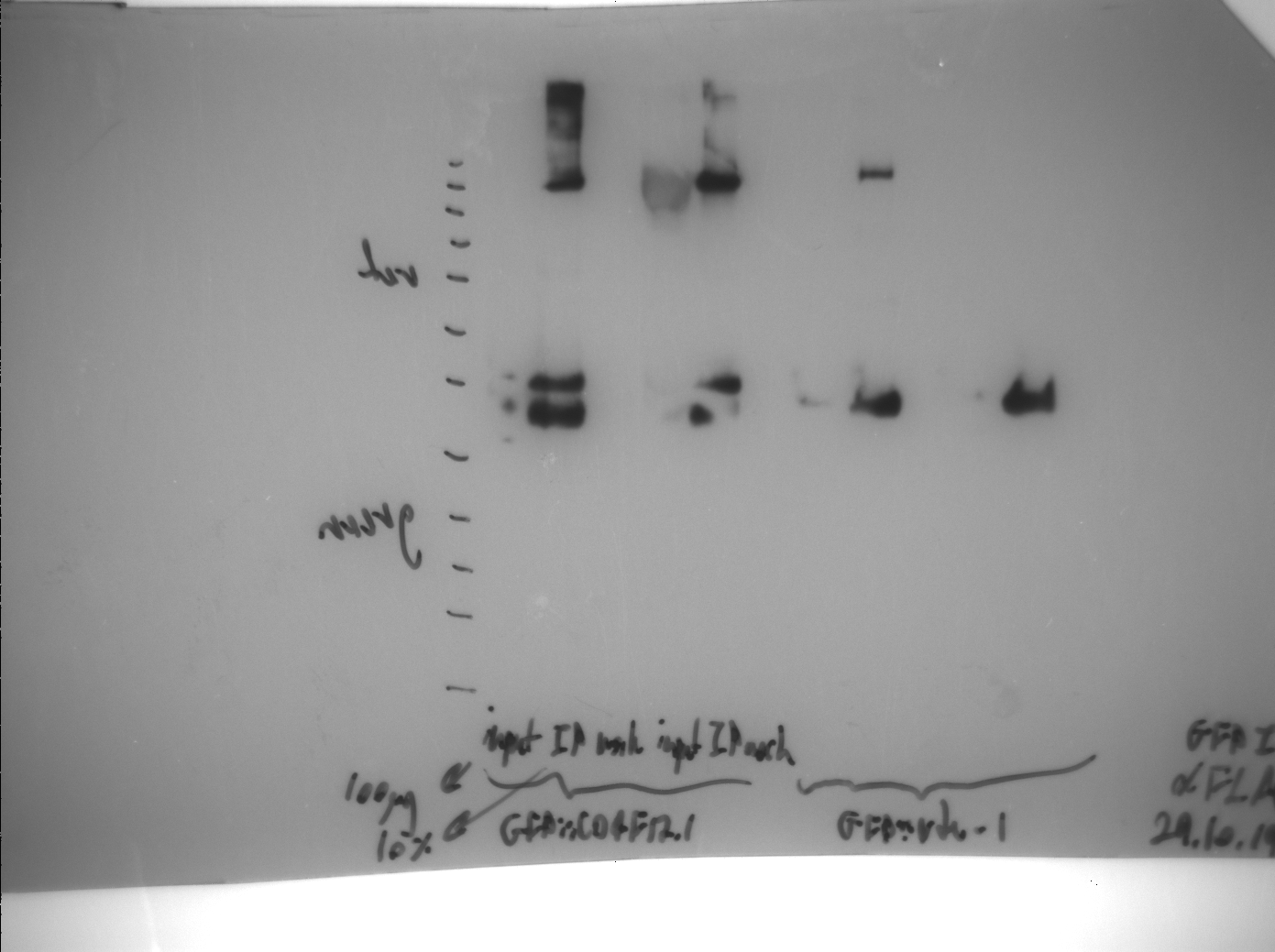

Supplement: Figure 1—figure supplement 1—source data 1. [file elife-83853-fig1-figsupp1-data1.zip › Figure S1/F_GFP-3xFLAG_C04F12.1_RDE-1.tif]

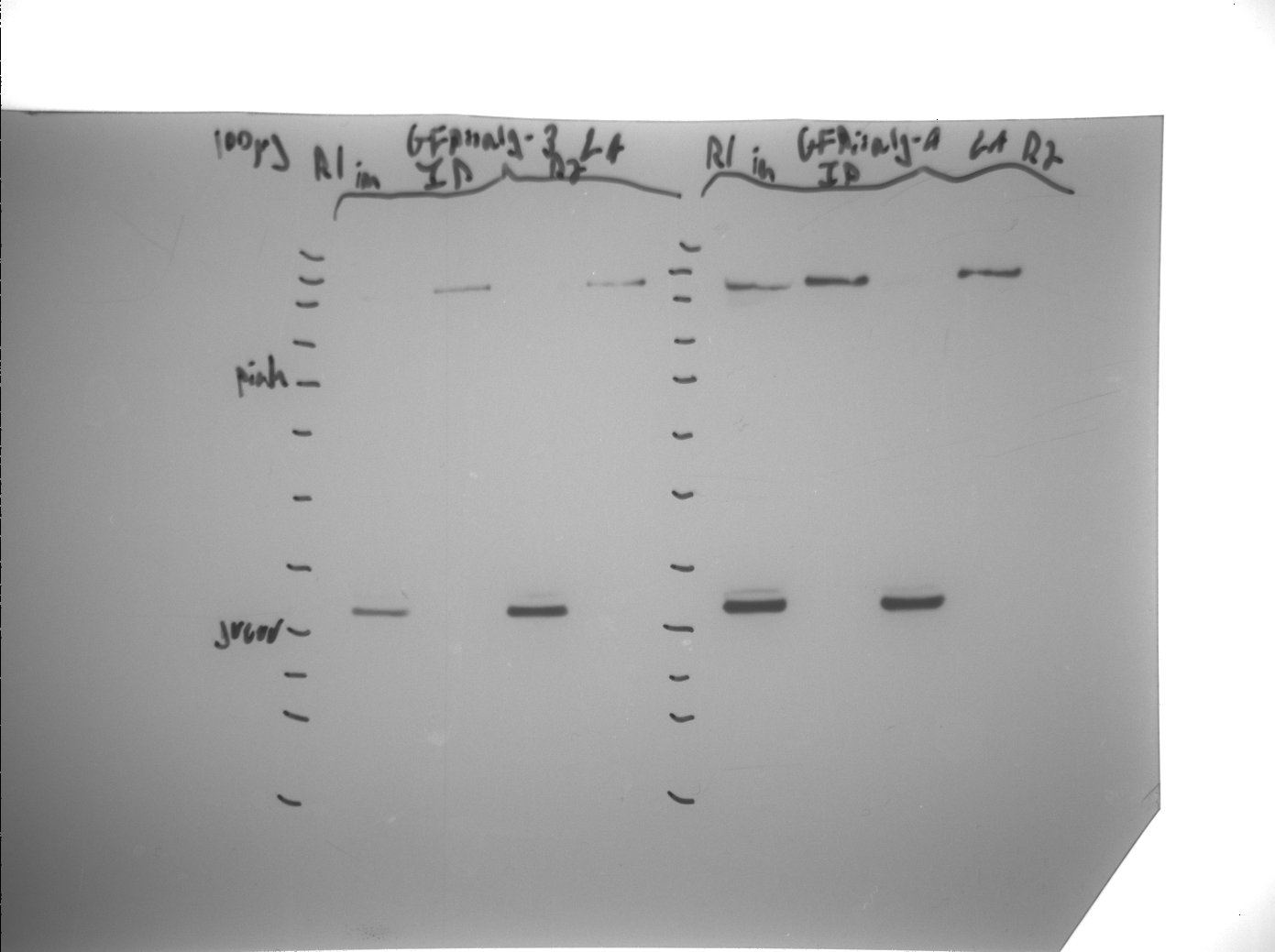

Supplement: Figure 1—figure supplement 1—source data 1. [file elife-83853-fig1-figsupp1-data1.zip › Figure S1/C_GFP-3xFLAG_ALG-3_ALG-4_L4.tif]

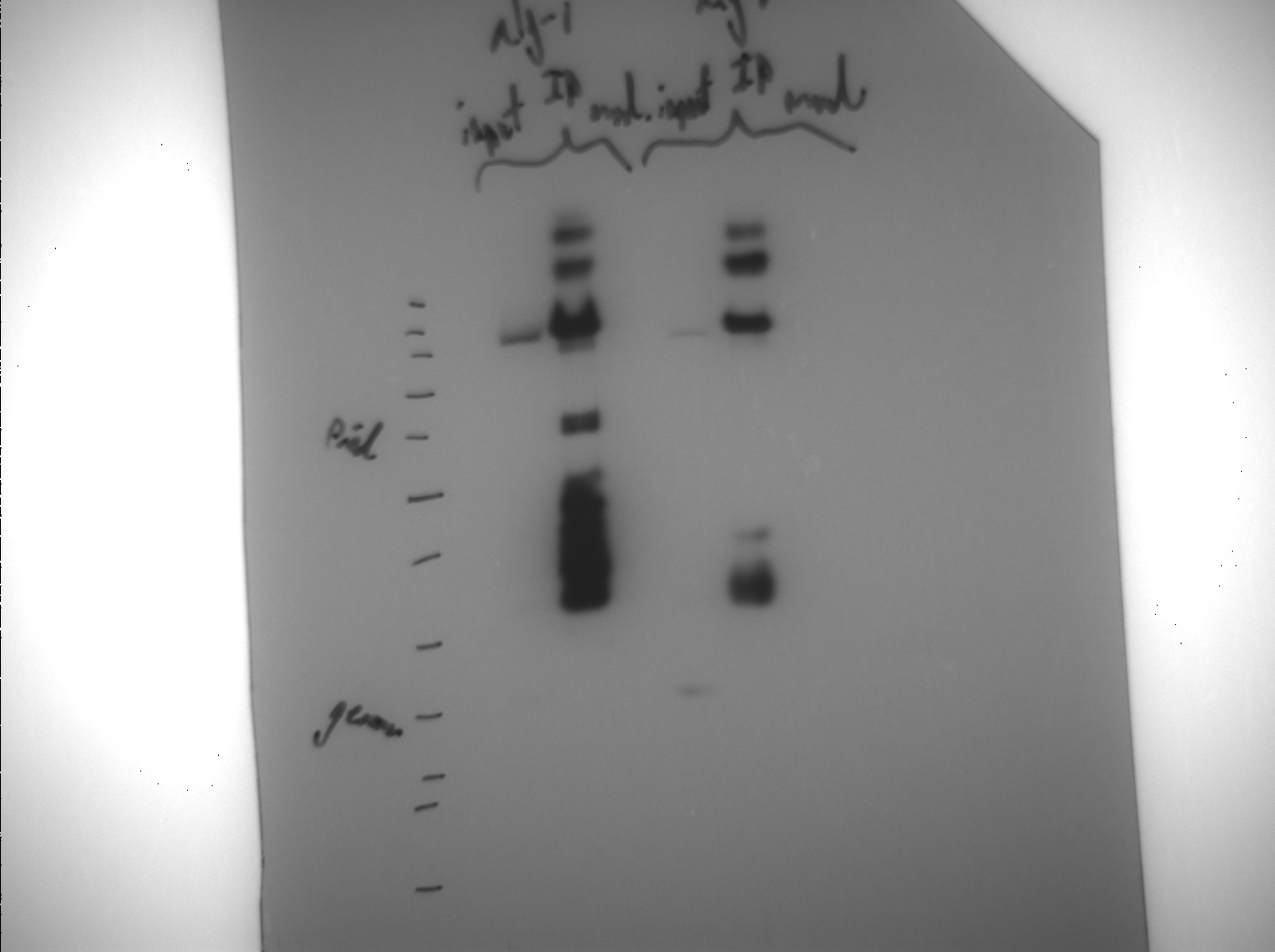

Supplement: Figure 1—figure supplement 1—source data 1. [file elife-83853-fig1-figsupp1-data1.zip › Figure S1/A_GFP-3xFLAG_ALG-1_ALG-2.tif]

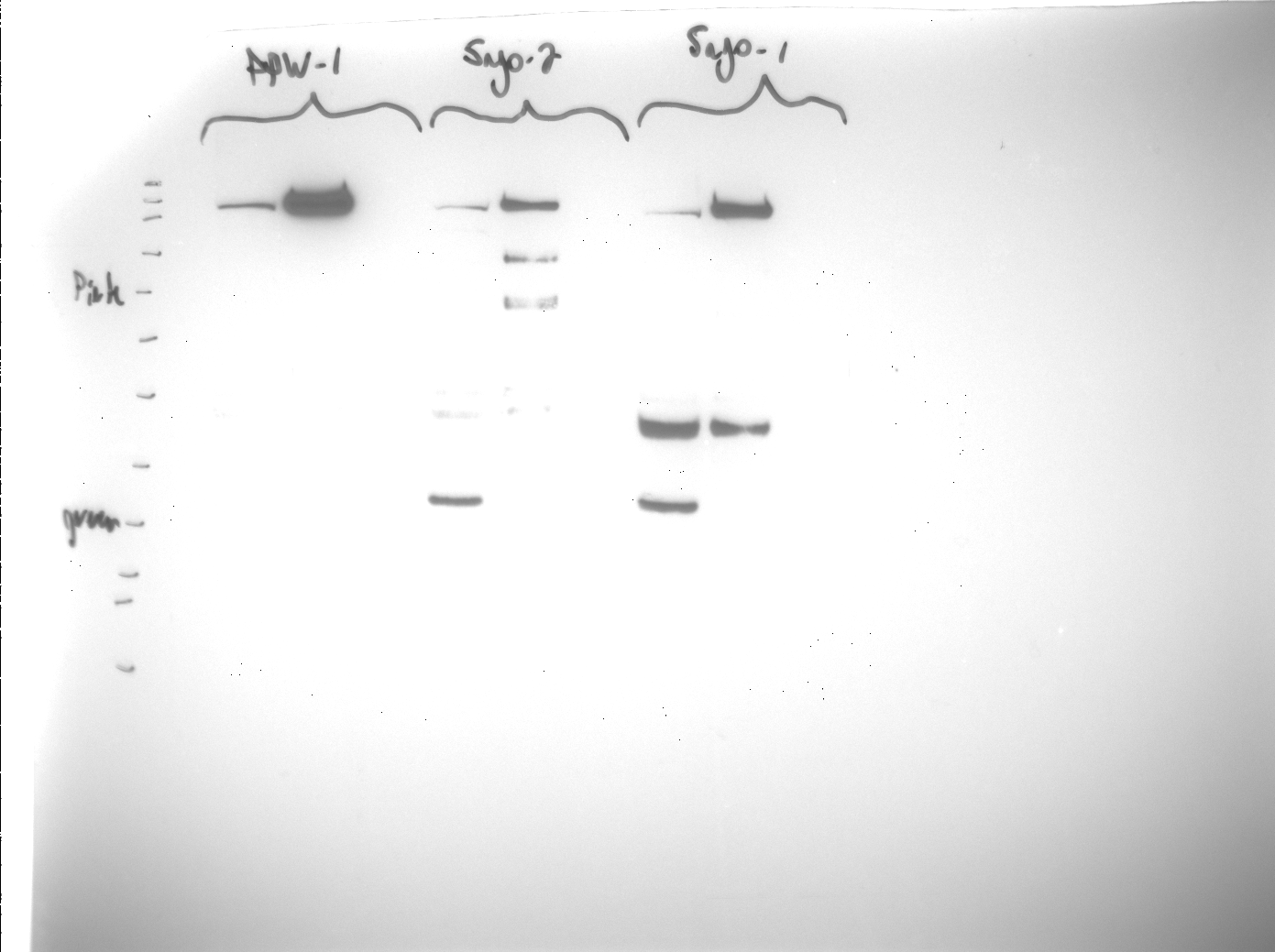

Supplement: Figure 1—figure supplement 1—source data 1. [file elife-83853-fig1-figsupp1-data1.zip › Figure S1/H_GFP-3xFLAG_PPW-1_SAGO-2_SAGO-1_2.tif]

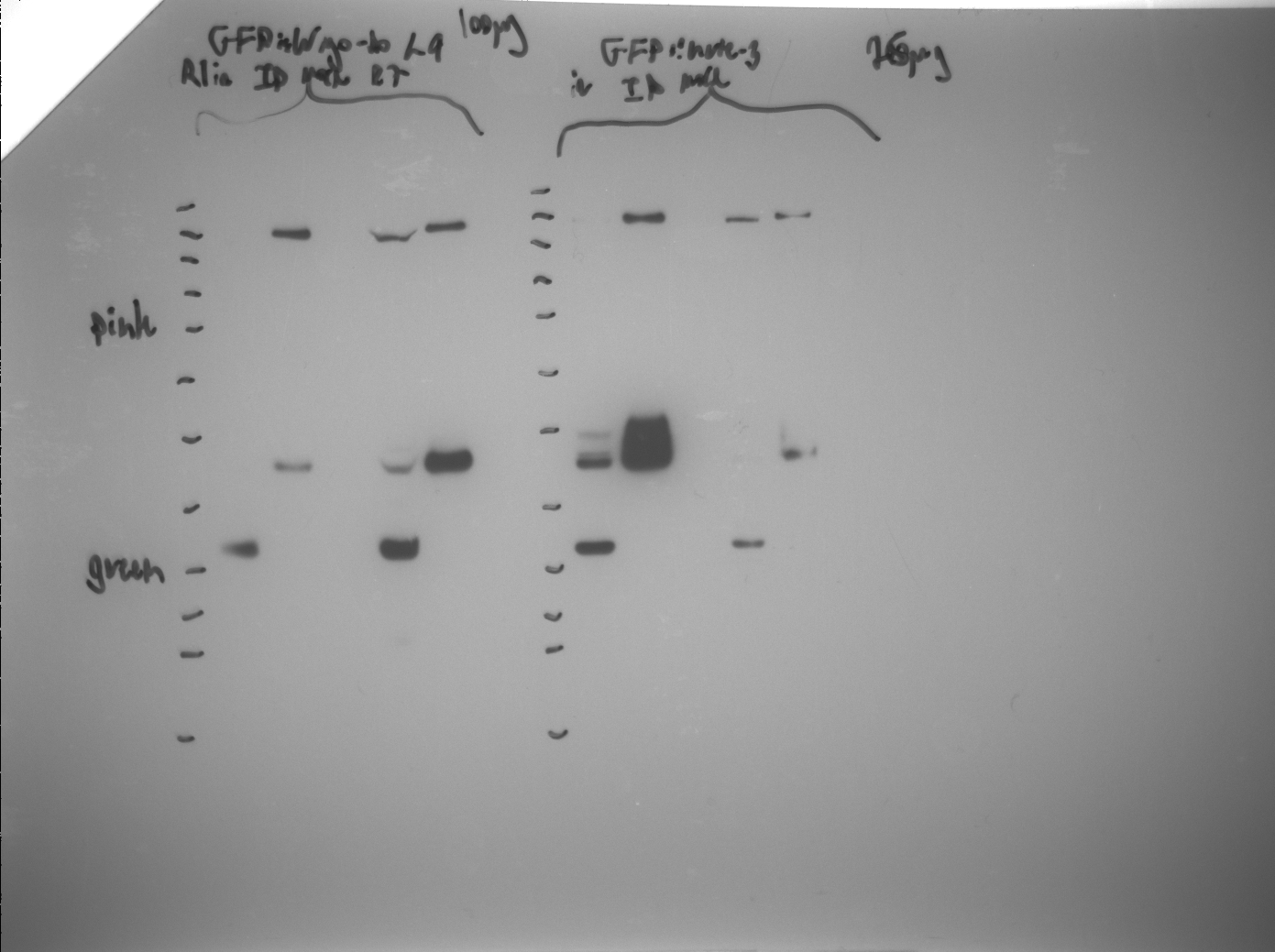

Supplement: Figure 1—figure supplement 1—source data 1. [file elife-83853-fig1-figsupp1-data1.zip › Figure S1/I_J_GFP-3xFLAG_WAGO-10_NRDE-3.tif]

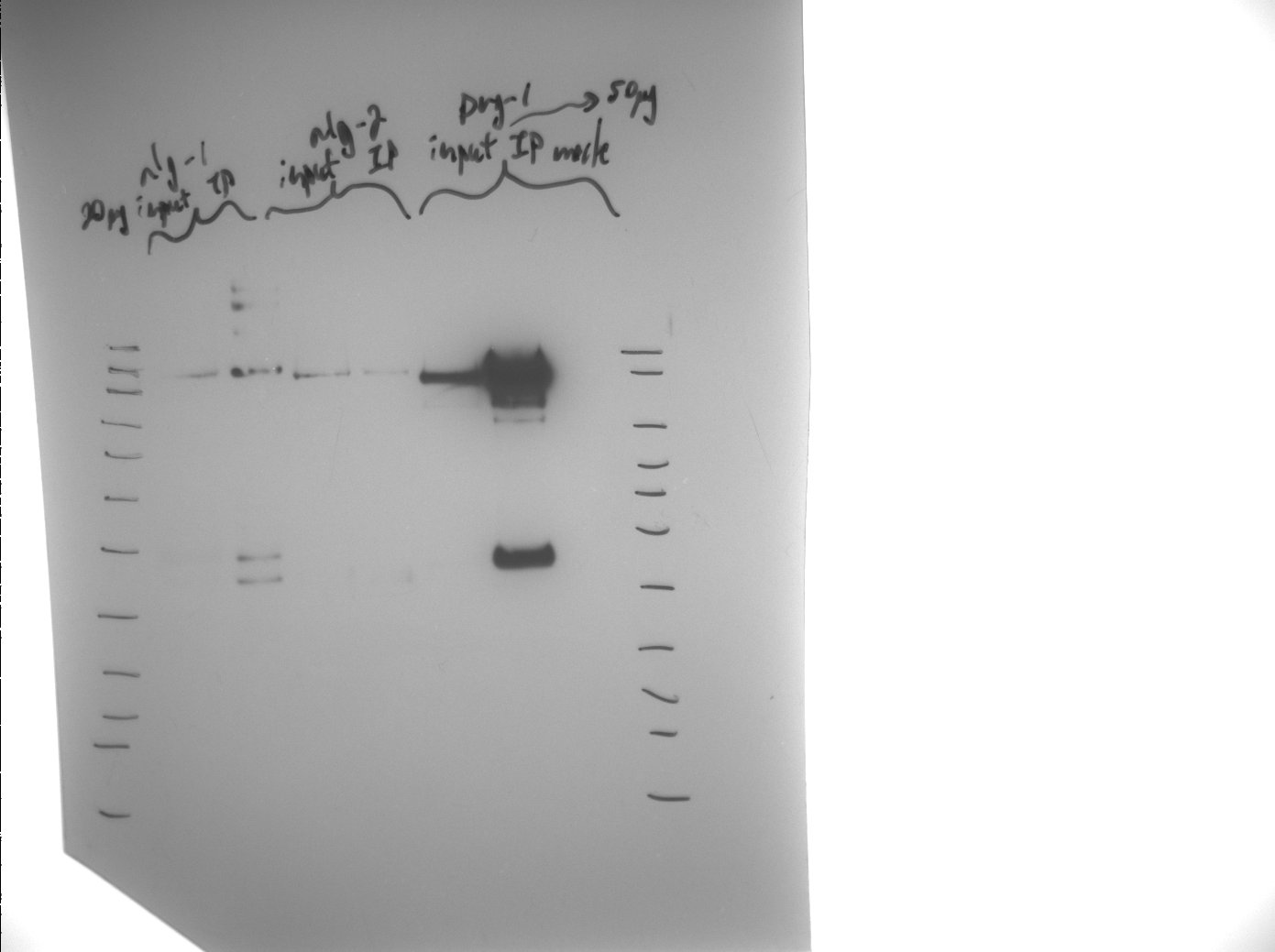

Supplement: Figure 1—figure supplement 1—source data 1. [file elife-83853-fig1-figsupp1-data1.zip › Figure S1/E_GFP-3xFLAG_ALG-1_ALG-2_PRG-1.tif]

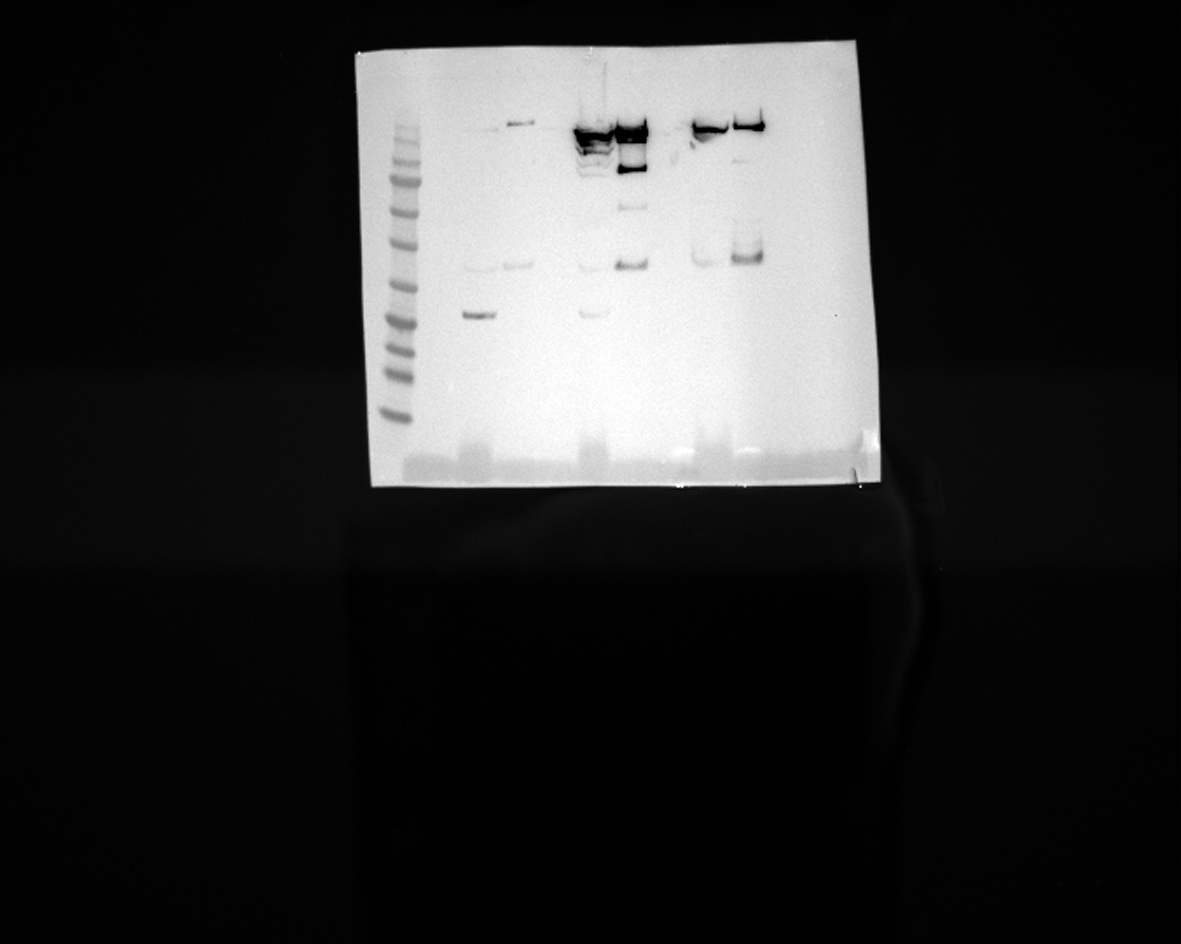

Supplement: Figure 1—figure supplement 1—source data 1. [file elife-83853-fig1-figsupp1-data1.zip › Figure S1/D_GFP-3xFLAG_RDE-1_CSR-1_WAGO-1.tif]

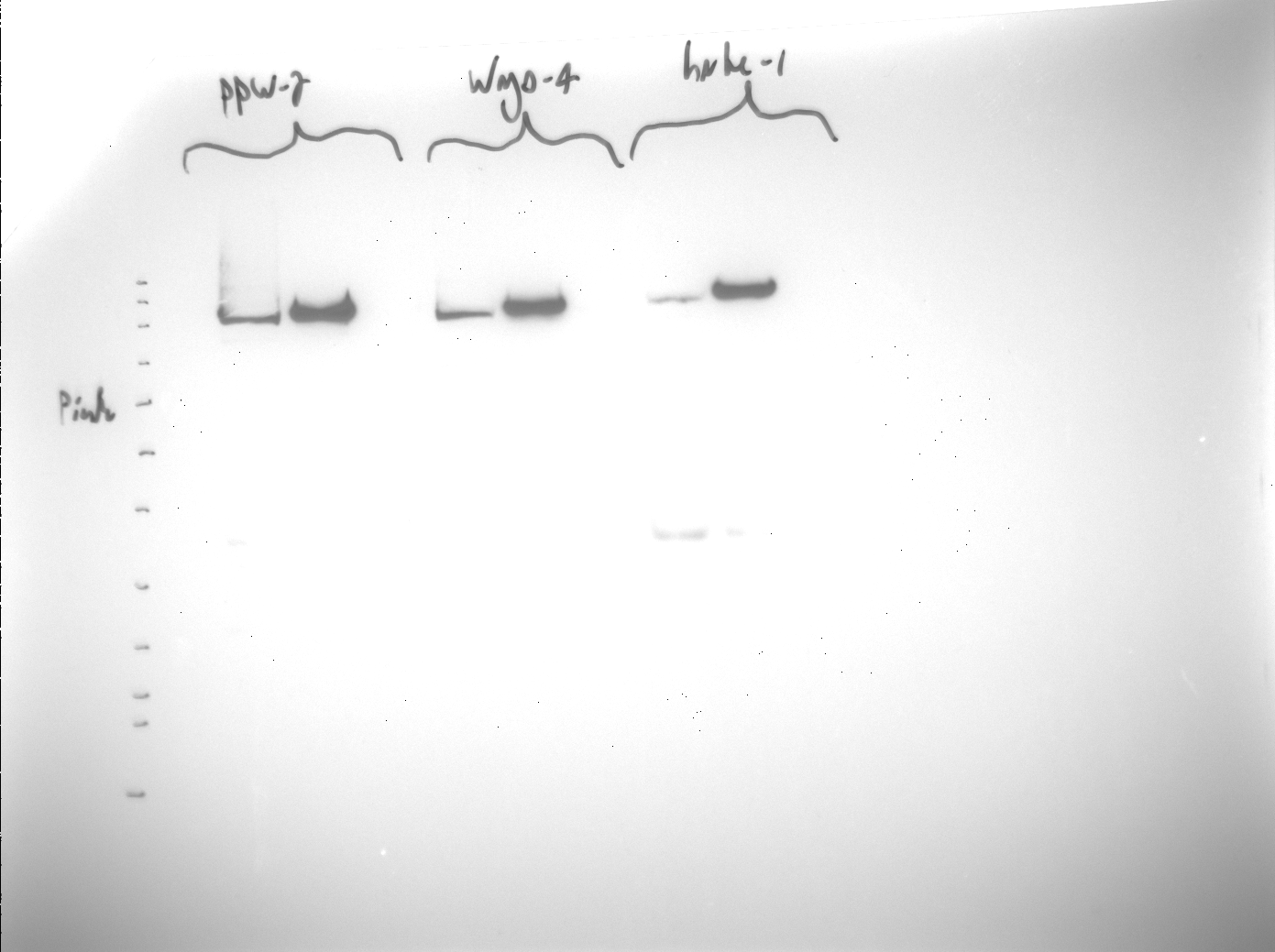

Supplement: Figure 1—figure supplement 1—source data 1. [file elife-83853-fig1-figsupp1-data1.zip › Figure S1/G_GFP-3xFLAG-PPW-2_WAGO-4_HRDE-1.tif]

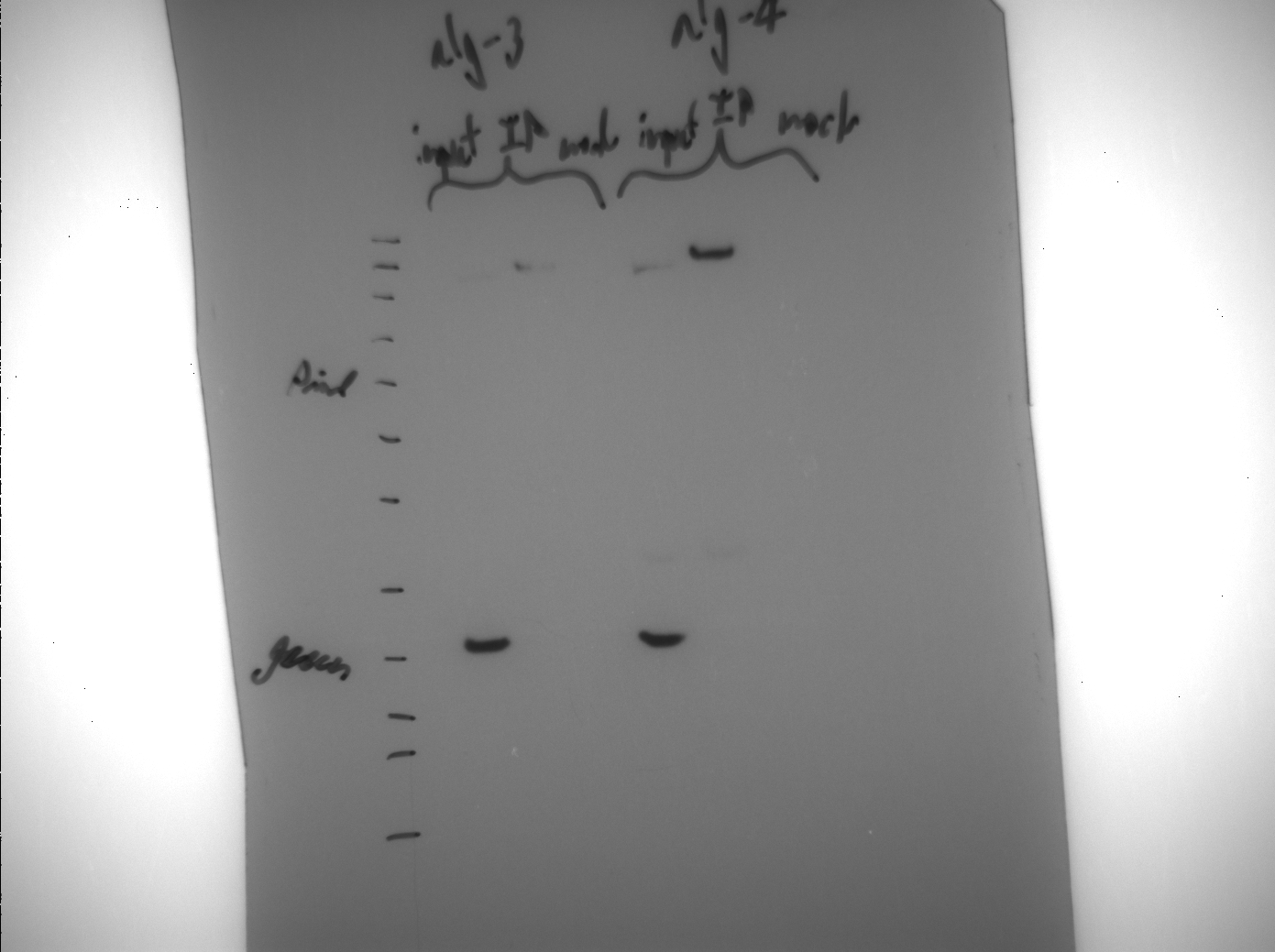

Supplement: Figure 1—figure supplement 1—source data 1. [file elife-83853-fig1-figsupp1-data1.zip › Figure S1/C_GFP-3xFLAG_ALG-3_ALG-4_L4_2.tif]

A

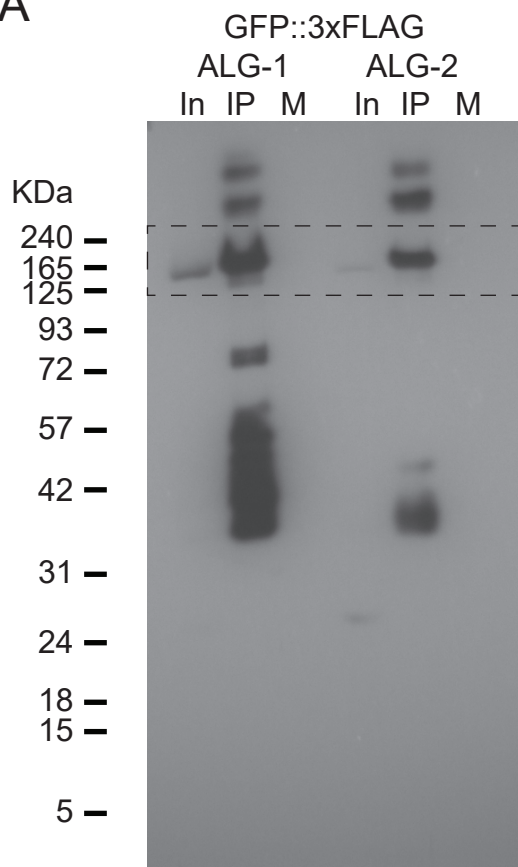

B

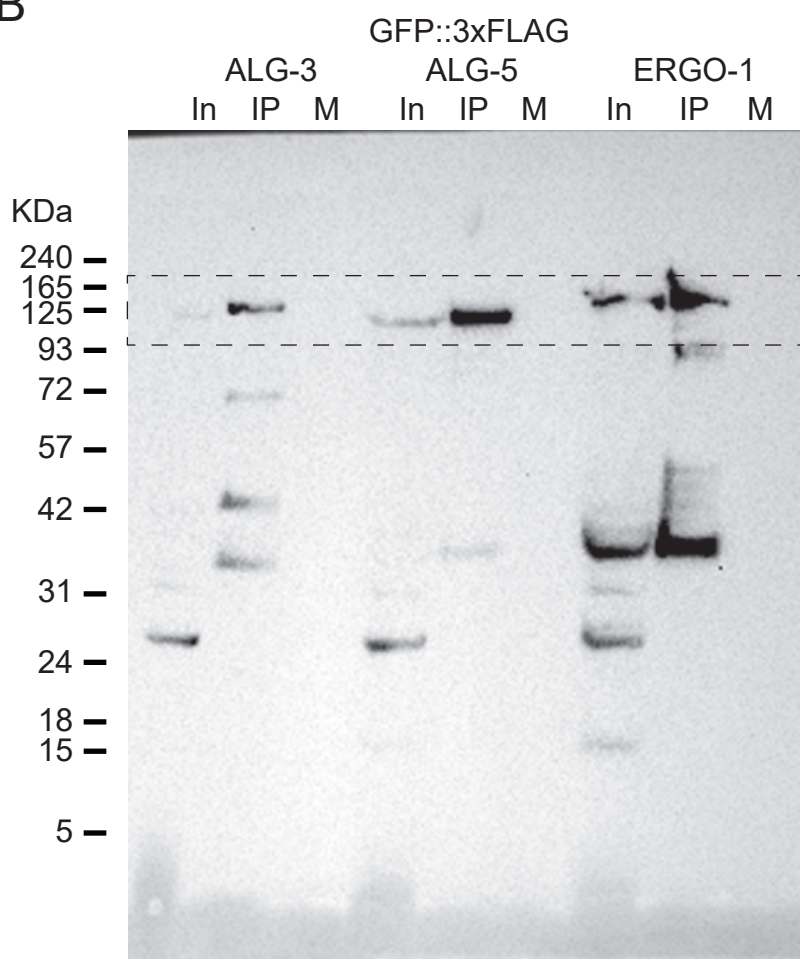

C

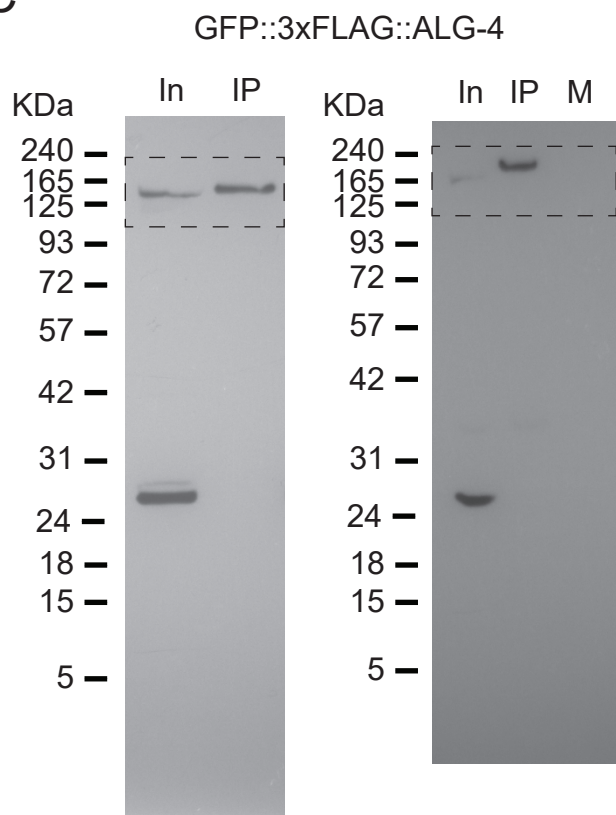

D

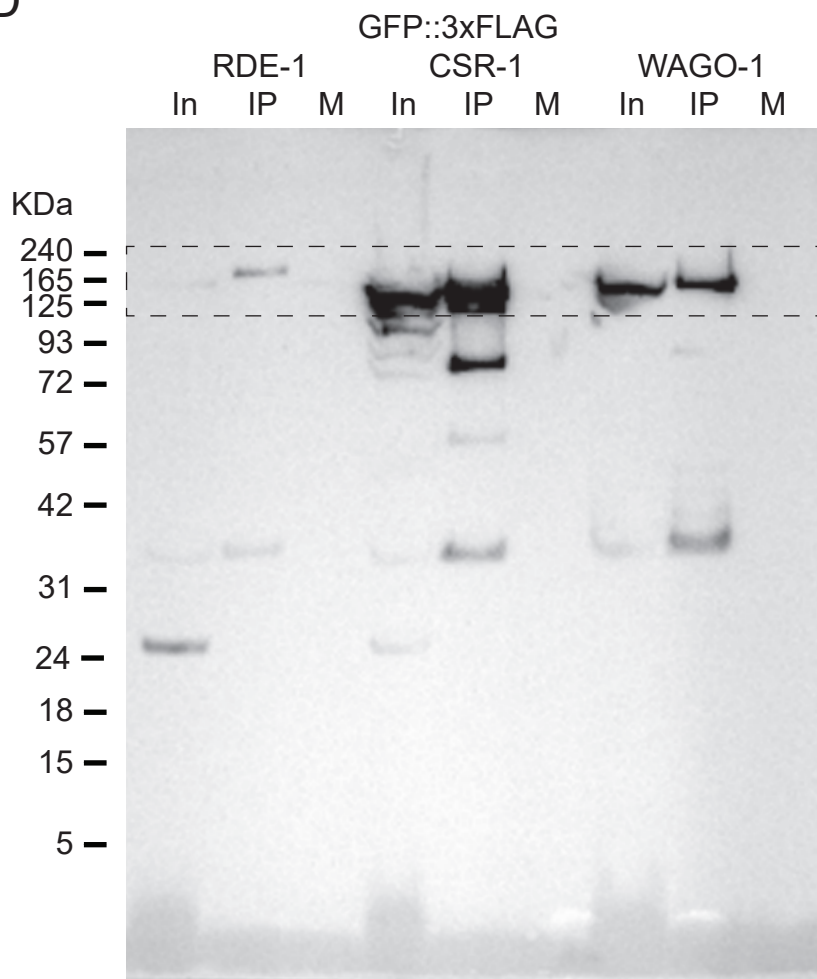

Supplement: Figure 1—figure supplement 1—source data 1. [file elife-83853-fig1-figsupp1-data1.zip › Figure S1/Figure S1 Blots.pdf]

E

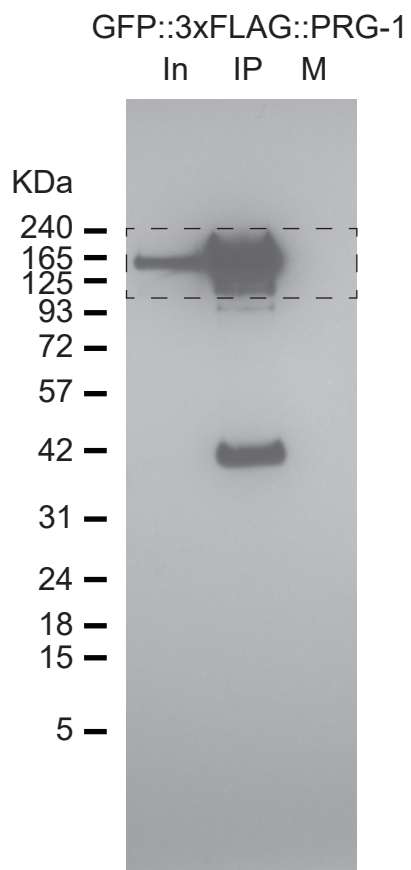

F

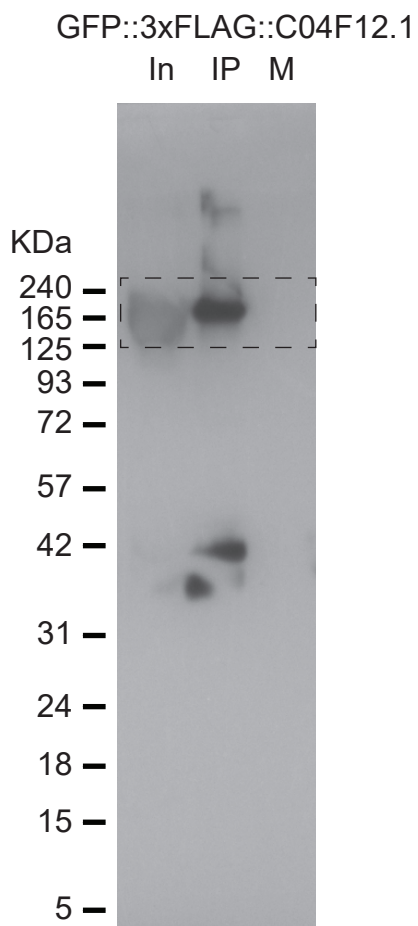

G

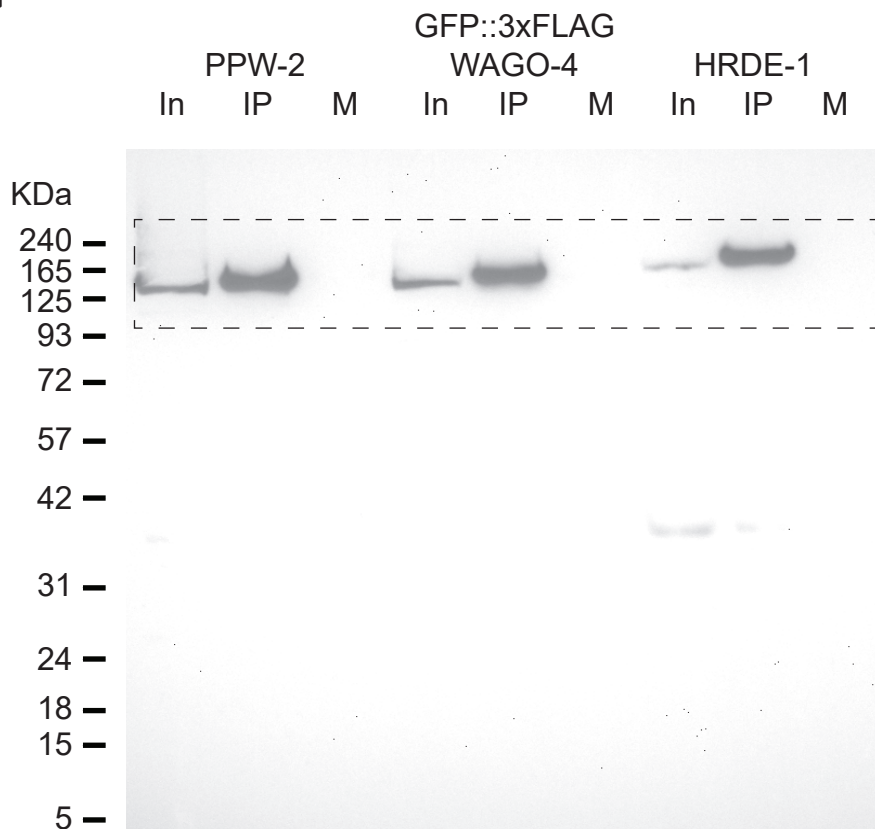

Supplement: Figure 1—figure supplement 1—source data 1. [file elife-83853-fig1-figsupp1-data1.zip › Figure S1/Figure S1 Blots 2.pdf]

H

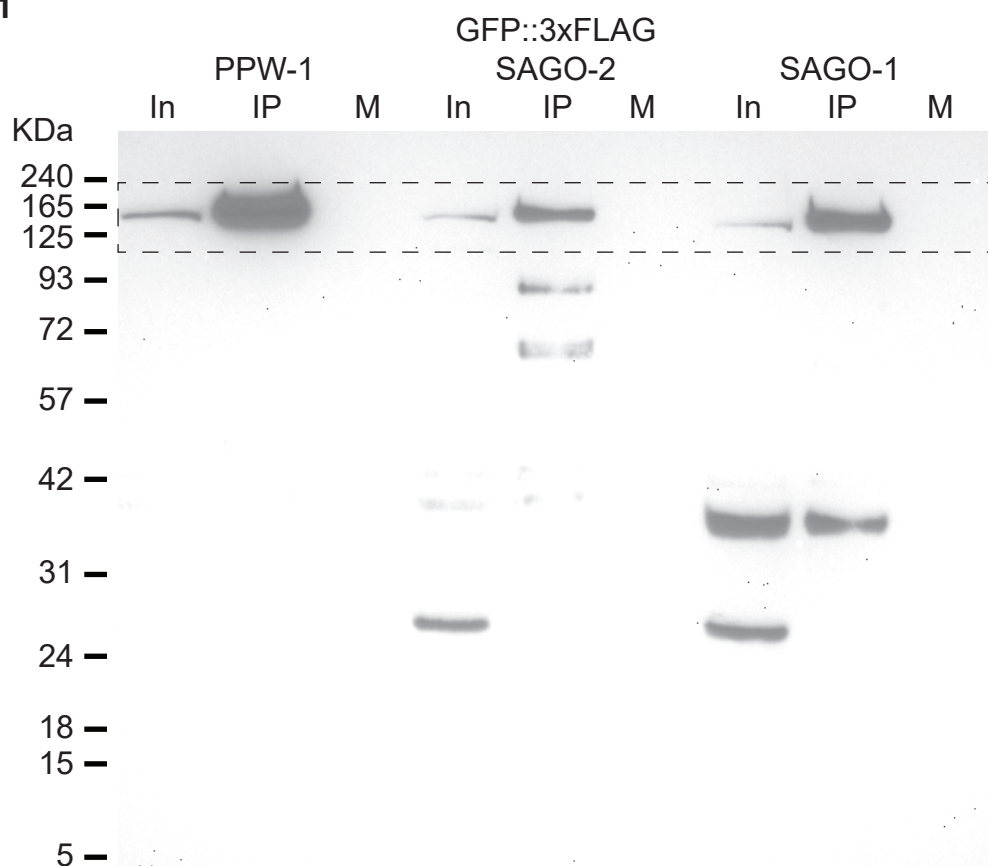

I

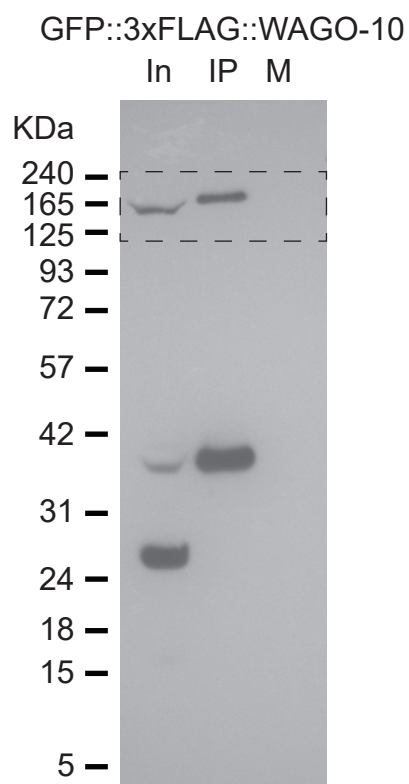

J

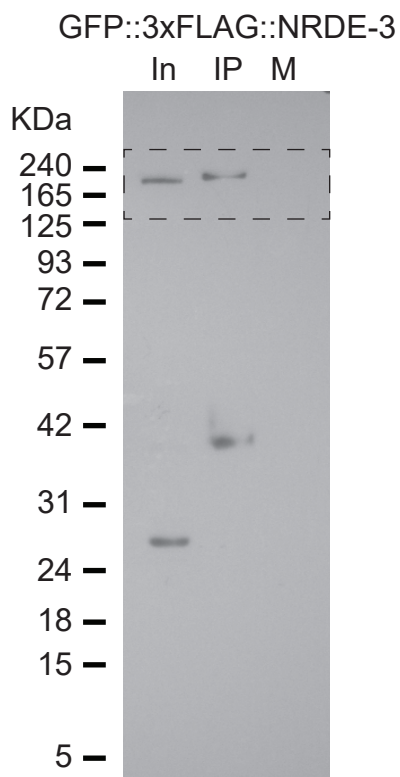

Supplement: Figure 1—figure supplement 1—source data 1. [file elife-83853-fig1-figsupp1-data1.zip › Figure S1/Figure S1 Blots 3.pdf]

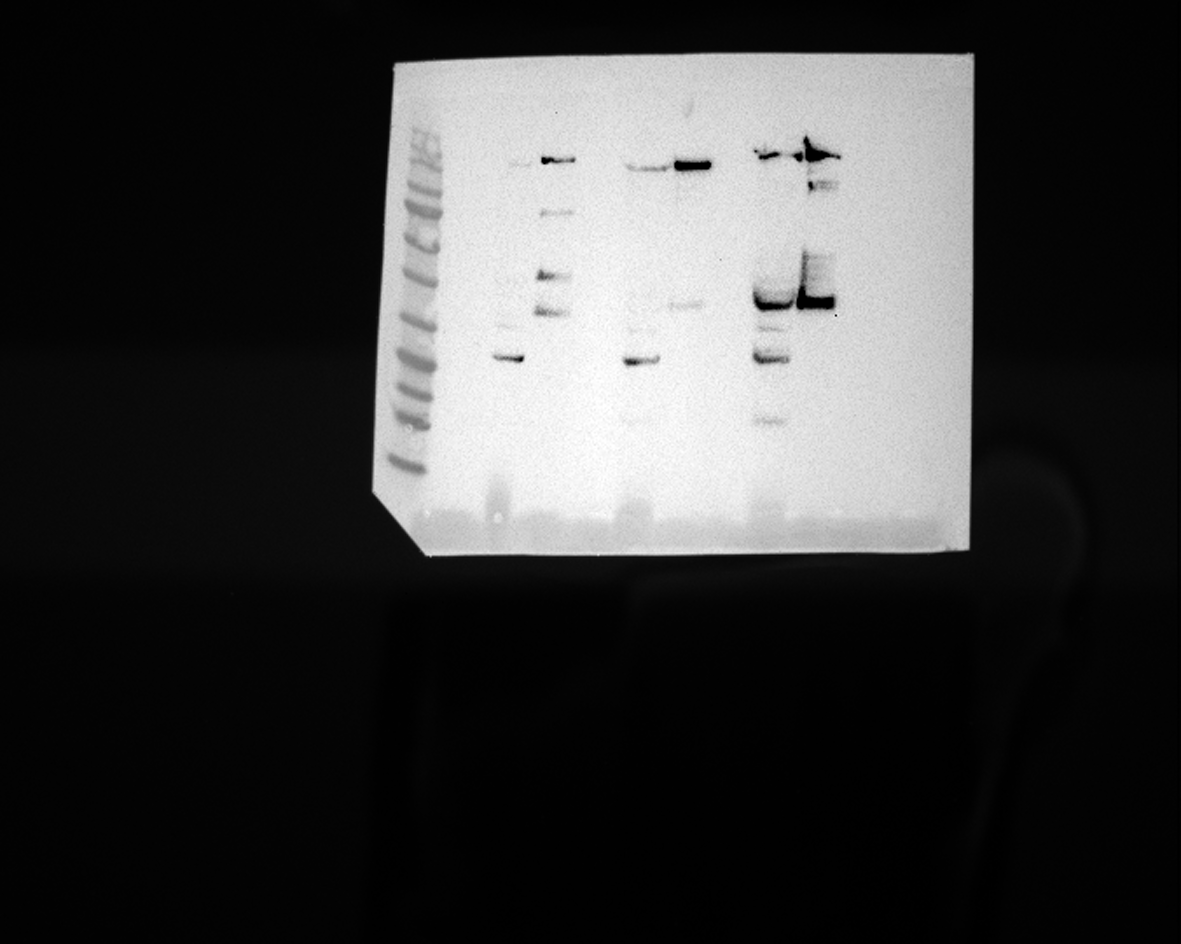

Supplement: Figure 1—figure supplement 1—source data 1. [file elife-83853-fig1-figsupp1-data1.zip › Figure S1/B_GFP-3xFLAG_ALG-3_ALG-5_ERGO-1.tif]

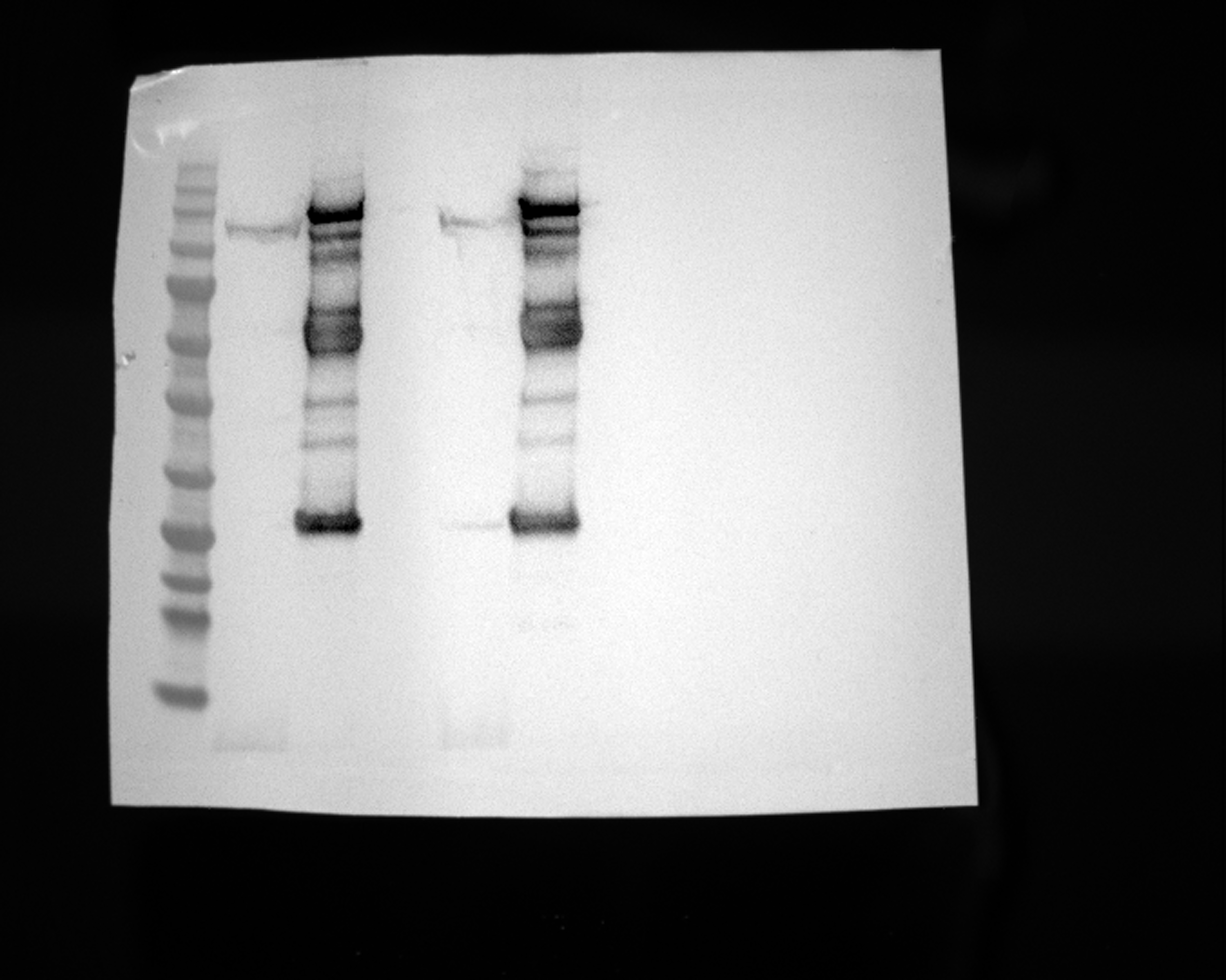

Supplement: Figure 1—figure supplement 2—source data 1. [file elife-83853-fig1-figsupp2-data1.zip › Figure S2/A_3xFLAG-C04F12.1.tif]

**A**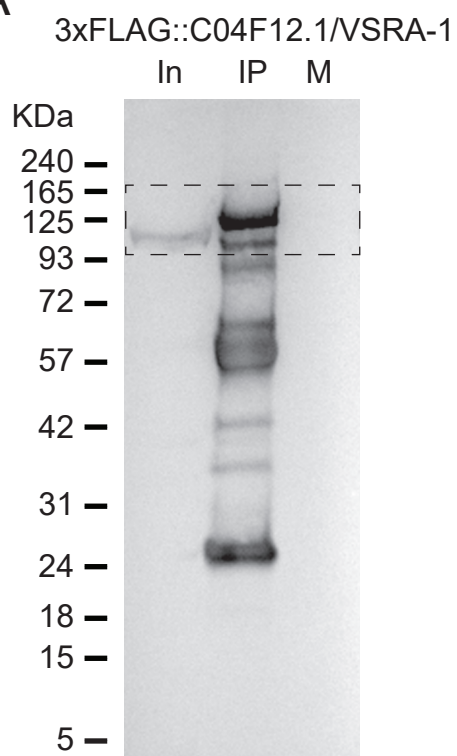**B**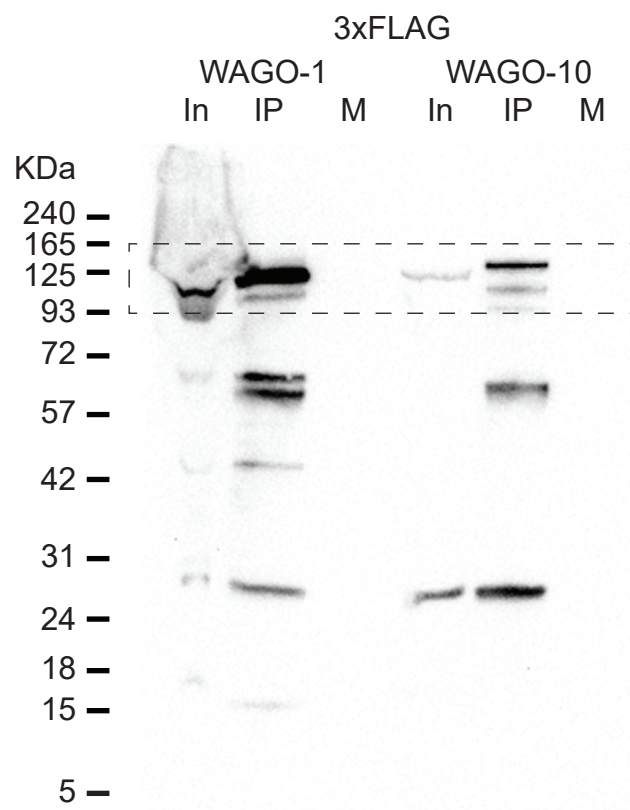**C**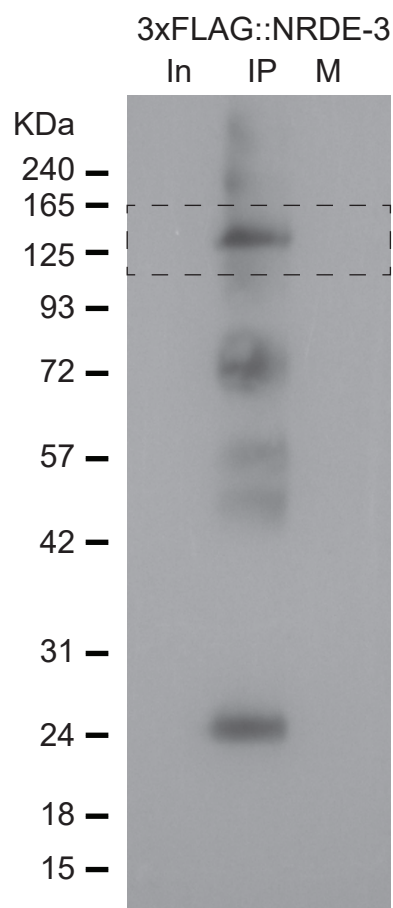

Supplement: Figure 1—figure supplement 2—source data 1. [file elife-83853-fig1-figsupp2-data1.zip › Figure S2/Figure S2 Blots.pdf]

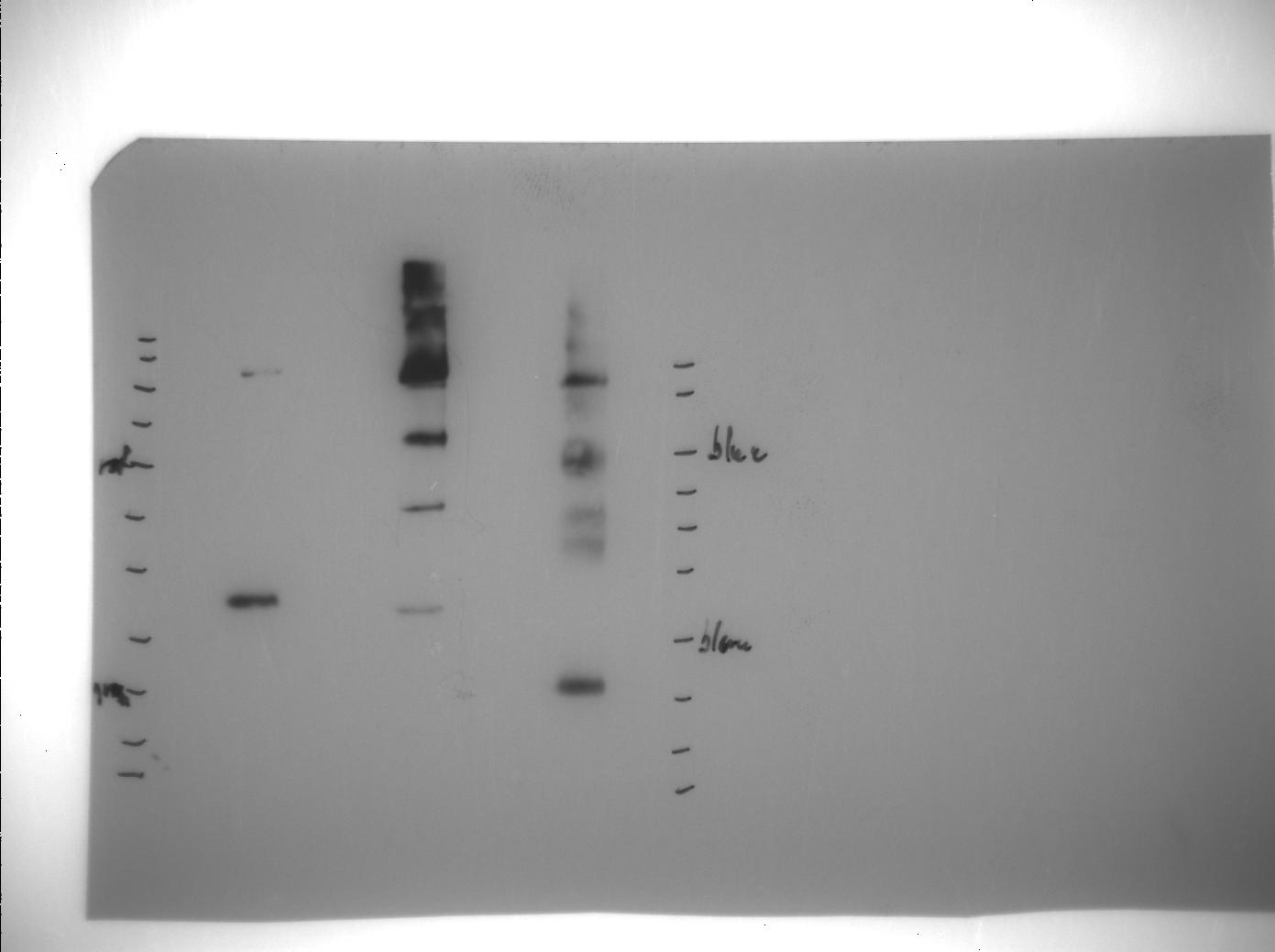

Supplement: Figure 1—figure supplement 2—source data 1. [file elife-83853-fig1-figsupp2-data1.zip › Figure S2/C_3xFLAG-GFP_ALG-5_CSR-1_3xFLAG_NRDE-3.tif]

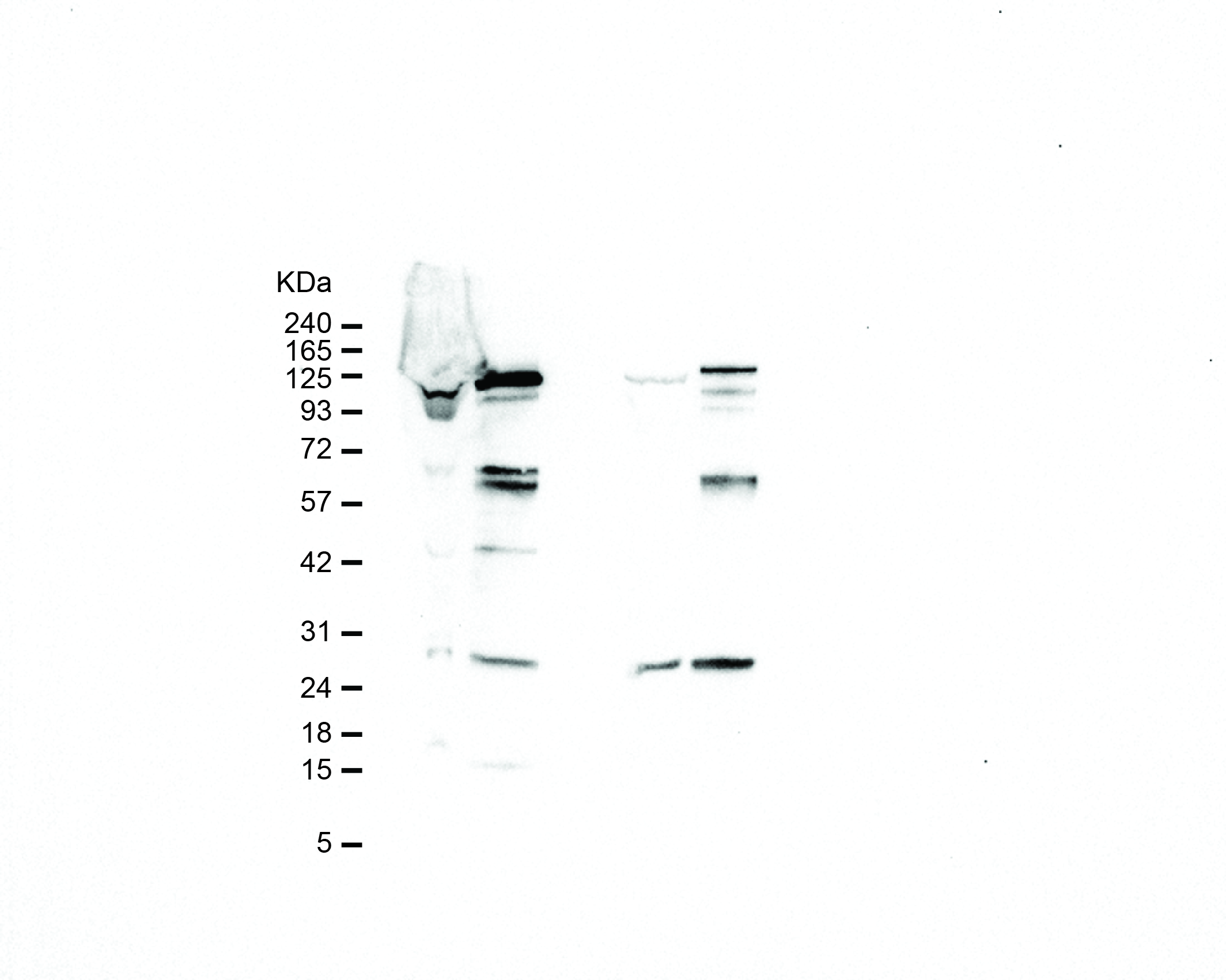

Supplement: Figure 1—figure supplement 2—source data 1. [file elife-83853-fig1-figsupp2-data1.zip › Figure S2/B_3xFLAG_WAGO-1_WAGO-10.jpg]

A

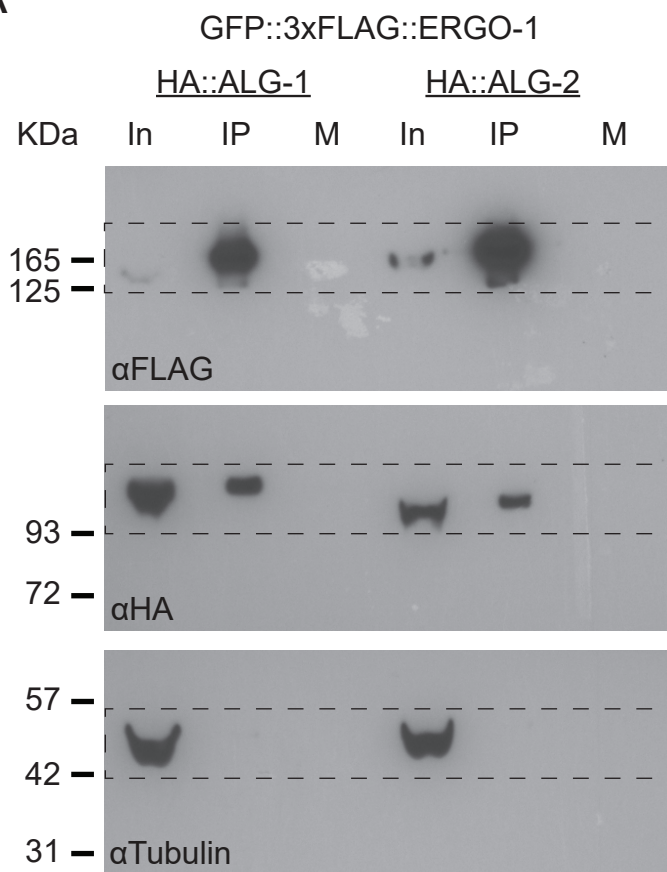

B

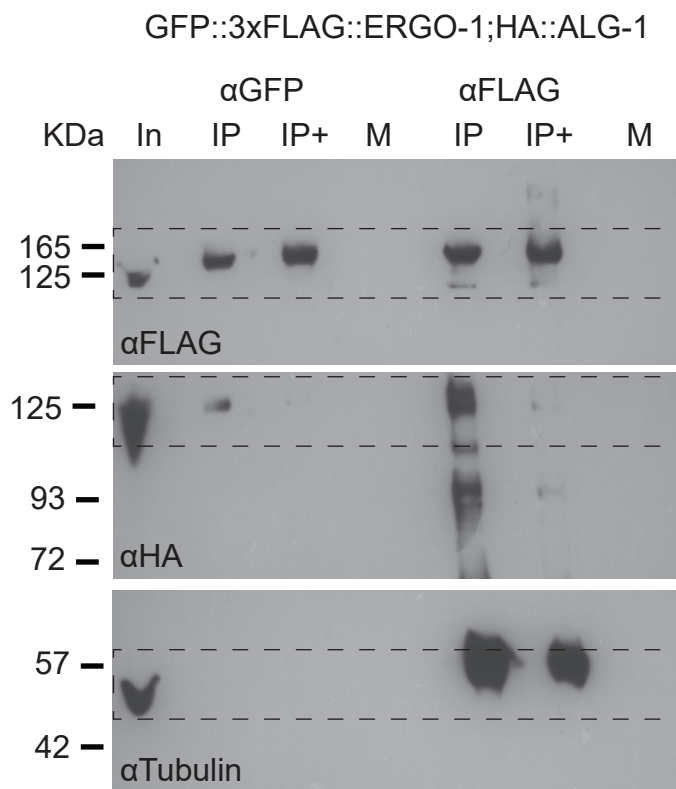

Supplement: Figure 3—source data 1. [file elife-83853-fig3-data1.zip › Figure 3/Figure 3 Blots.pdf]

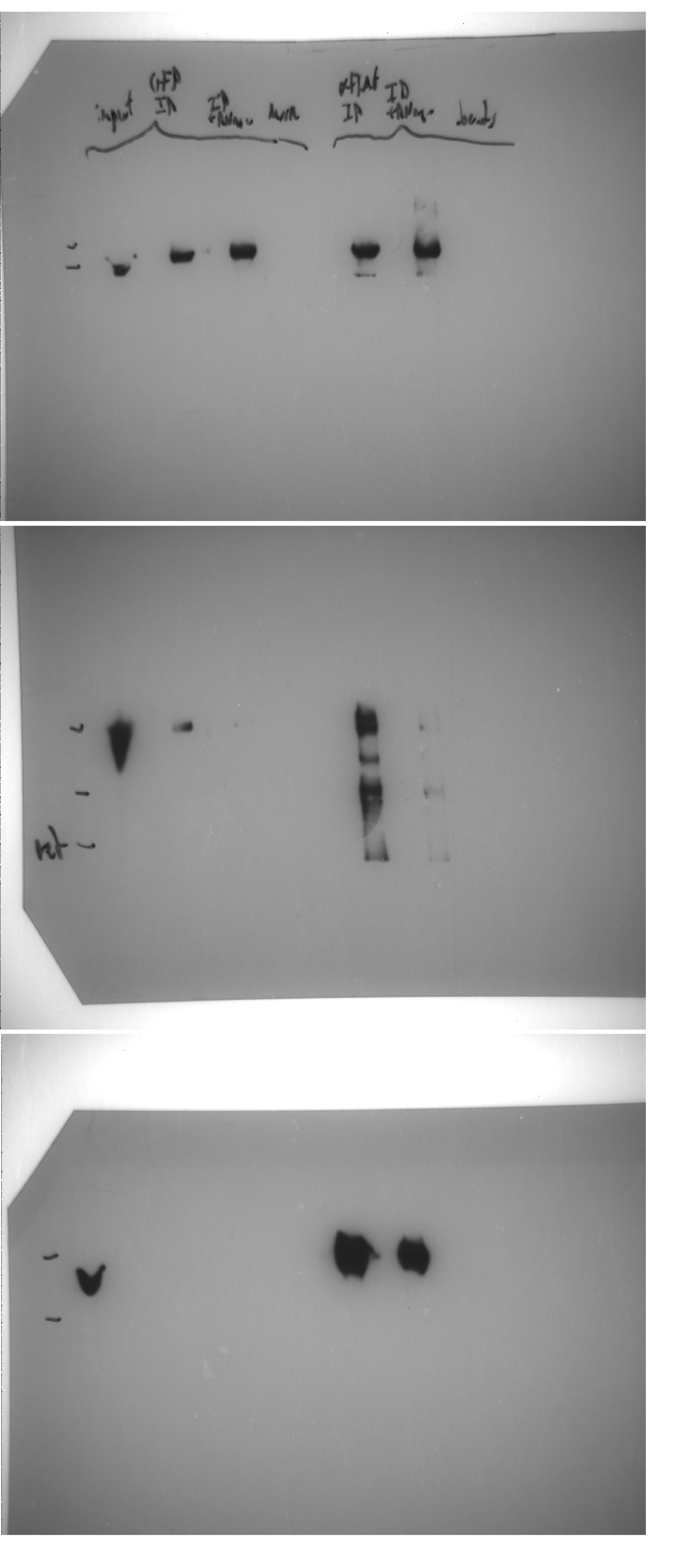

Supplement: Figure 3—source data 1. [file elife-83853-fig3-data1.zip › Figure 3/B_GFP-3xFLAG-ERGO-1_HA-ALG-1_RNASE.jpg]

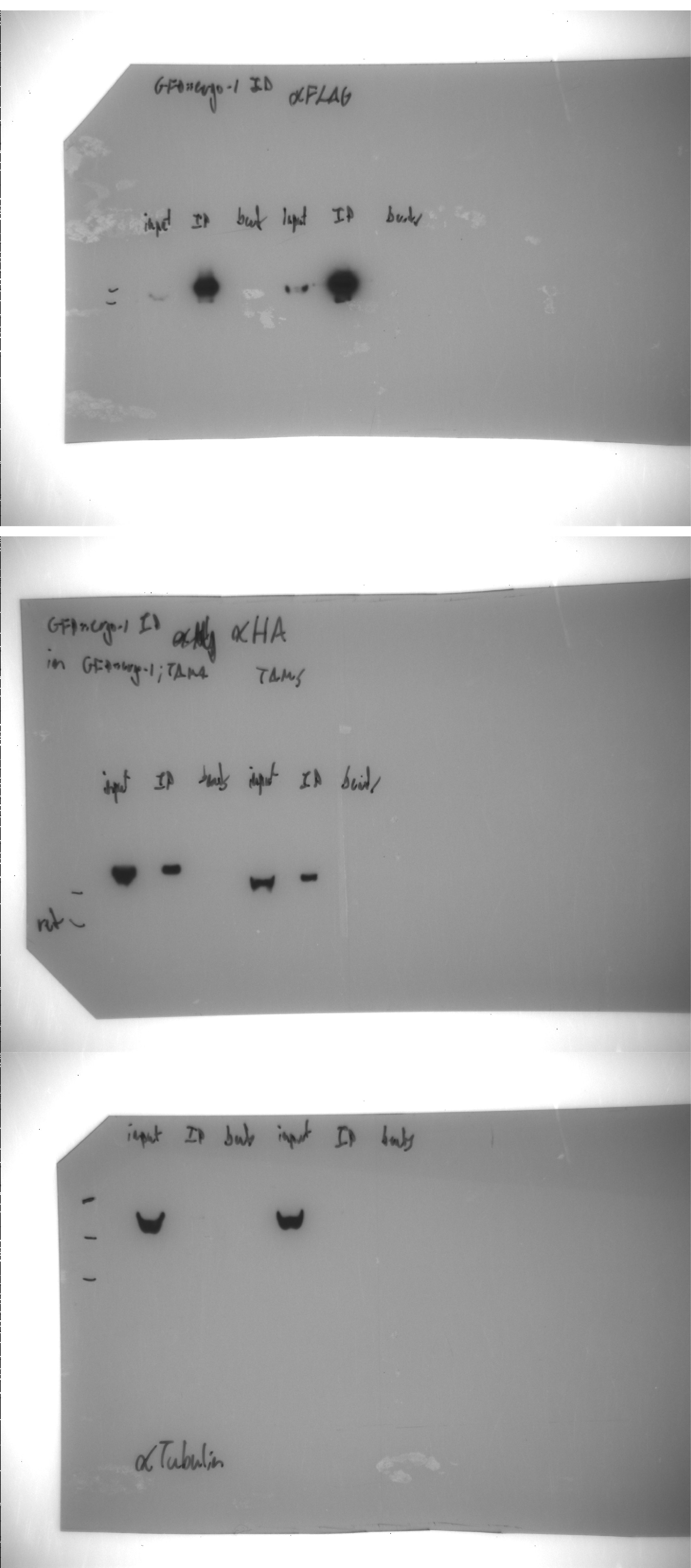

Supplement: Figure 3—source data 1. [file elife-83853-fig3-data1.zip › Figure 3/A_GFP-3xFLAG-ERGO-1_HA-ALG-1_HA-ALG-2.jpg]
