# Supplementary material for: A comprehensive survey of C. elegans argonaute proteins reveals organism-wide gene regulatory networks and functions
Source: eLife. 2023 Feb 15;12:e83853. doi: 10.7554/eLife.83853 (PMC10101689; doi:10.7554/eLife.83853)
Supplement: Figure 1—figure supplement 2—source data 1. [file elife-83853-fig1-figsupp2-data1.zip › Figure S2/Figure S2 Blots Legend.docx]

A.

Original File: A_3xFLAG-C04F12.1.tif

3xFLAG::C04F12.1/VSRA-1 IP (mouse anti-FLAG M2 antibody)/WB (mouse anti-FLAG M2 antibody)

Blot from Figure S2E

Synchronized YA hermaphrodite samples

In = Input (total lysate) 100ug

IP = Immunoprecipitation with mouse anti-FLAG M2 antibody beads 10% of 5mg IP

M = Immunoprecipitation with non-specific antibody beads 10% of 5mg IP

MW of 3xFLAG::C04F12.1/VSRA-1 ~ 110kDa

B.

Original File: B_3xFLAG_WAGO-1_WAGO-10.jpg

3xFLAG::WAGO-1 IP (mouse anti-FLAG M2 antibody)/WB (mouse anti-FLAG M2 antibody)

3xFLAG::WAGO-10 IP (mouse anti-FLAG M2 antibody)/WB (mouse anti-FLAG M2 antibody)

Blot from Figure S2E

Synchronized YA hermaphrodite samples (WAGO-1)

Synchronized L4 hermaphrodite samples (WAGO-10)

In = Input (total lysate) 100ug

IP = Immunoprecipitation with mouse anti-FLAG M2 antibody beads 10% of 5mg IP

M = Immunoprecipitation with non-specific antibody beads 10% of 5mg IP

MW of 3xFLAG::WAGO-1 ~ 110kDa

MW of 3xFLAG::WAGO-10 ~ 110kDa

C.

Original File: C_3xFLAG-GFP_ALG-5_CSR-1_3xFLAG_NRDE-3.tif

3xFLAG::NRDE-3 IP (mouse anti-FLAG M2 antibody)/WB (mouse anti-FLAG M2 antibody)

Blot from Figure S2E

Synchronized YA hermaphrodite samples

In = Input (total lysate) 100ug

IP = Immunoprecipitation with mouse anti-FLAG M2 antibody beads 10% of 5mg IP

M = Immunoprecipitation with non-specific antibody beads 10% of 5mg IP

MW of 3xFLAG::NRDE-3 ~ 120kDa
