# Supplementary material for: A comprehensive survey of C. elegans argonaute proteins reveals organism-wide gene regulatory networks and functions
Source: eLife. 2023 Feb 15;12:e83853. doi: 10.7554/eLife.83853 (PMC10101689; doi:10.7554/eLife.83853)
Supplement: Figure 3—source data 1. [file elife-83853-fig3-data1.zip › Figure 3/Figure 3 Blots Legend.docx]

A.

Original File: A_GFP-3xFLAG-ERGO-1_HA-ALG-1_HA-ALG-2.jpg

GFP::3xFLAG::ERGO-1;HA::ALG-1 IP (Chromotek anti-GFP nanobodies)/WB (mouse anti-FLAG M2 antibody, Millipore Rat Anti-HA High Affinity, Sigma Mouse anti-alpha Tubulin)

GFP::3xFLAG::ERGO-1;HA::ALG-2 IP (Chromotek anti-GFP nanobodies)/WB (mouse anti-FLAG M2 antibody, Millipore Rat Anti-HA High Affinity, Sigma Mouse anti-alpha Tubulin)

Blot from Figure 3E

Synchronized YA hermaphrodite samples

In = Input (total lysate) 100ug

IP = Immunoprecipitation with anti-GFP beads 100% of 5mg IP

M = Immunoprecipitation with non-specific antibody beads 100% of 5mg IP

MW of GFP:3xFLAG::ERGO-1 ~ 160kDa

MW of HA::ALG-1 ~ 110kDa

MW of HA::ALG-2 ~ 110kDa

MW of Tubulin ~ 45kDa

B.

Original File: B_GFP-3xFLAG-ERGO-1_HA-ALG-1_RNASE.jpg

GFP::3xFLAG::ERGO-1;HA::ALG-1 IP (Chromotek anti-GFP nanobodies)/WB (mouse anti-FLAG M2 antibody, Millipore Rat Anti-HA High Affinity, Sigma Mouse anti-alpha Tubulin)

GFP::3xFLAG::ERGO-1;HA::ALG-1 IP (mouse anti-FLAG M2 antibody)/WB (mouse anti-FLAG M2 antibody, Millipore Rat Anti-HA High Affinity, Sigma Mouse anti-alpha Tubulin)

Blot from Figure 3F

Synchronized YA hermaphrodite samples

In = Input (total lysate) 100ug

IP = Immunoprecipitation with anti-GFP beads 100% of 5mg IP

IP = Immunoprecipitation with anti-GFP beads 100% of 5mg IP + 100U RNase I (Ambion)

M = Immunoprecipitation with non-specific antibody beads 100% of 5mg IP

MW of GFP:3xFLAG::ERGO-1 ~ 160kDa

MW of HA::ALG-1 ~ 110kDa

MW of Tubulin ~ 45kDa
